# Exploring Perceptions of Leveraging AI to Improve Outcomes in Maternal, Sexual, and Reproductive Health in Sub-Saharan Africa: Exploratory Qualitative Study

**DOI:** 10.2196/84378

**Published:** 2026-08-13

**Authors:** Rachel King, Elizabeth Oseku, Cecilia Akatukwasa, Moreen Nanyonjo, Joshua Beinomugisha, Joan Akullo, Jackie Ssemata, Rosalind Parkes-Ratanshi

**Affiliations:** 1 Department of Epidemiology and Biostatistics, Institute for Global Health Sciences, University of California, San Francisco, 550 16th St., Floor 3, San Francisco, CA, 94143, United States, 1 415-476-5494; 2 Academy for Health Innovations, Infectious Diseases Institute, Kampala, Uganda; 3 School of Medicine, Dentistry and Biomedical Sciences, Queens University Belfast, Belfast, Ireland

**Keywords:** sub-Saharan Africa, AI, artificial intelligence, maternal health, sexual health, reproductive health

## Abstract

**Background:**

AI has the potential to transform health care in low- and middle-income countries, where access to quality care remains limited. Maternal, sexual, and reproductive health (MSRH) outcomes are especially poor due to resource shortages, financial barriers, and geographic inequities. With thoughtful implementation, AI could help address these gaps through innovations in diagnostics, health education chatbots, and telemedicine. However, responsible use is essential to ensure AI reduces, rather than exacerbates, health disparities between high- and low-income regions.

**Objective:**

Our study examines the perceptions, uses, benefits, and challenges of AI in MSRH among medical professionals, community members, and AI experts, guided by the diffusion of innovations theory.

**Methods:**

We conducted an exploratory qualitative study involving a round table discussion, key informant interviews, focus group discussions, and stakeholder meetings, to examine the perceptions of health workers, policymakers, AI researchers and implementers, as well as Community Advisory Board members. We explored the opportunities, risks, limitations, and best practices for responsible AI in MSRH in sub-Saharan Africa. Framework analysis was used to analyze the collected data, and 3 member-check sessions were conducted to verify the accuracy of the findings. Finally, the themes derived from the data were mapped onto the diffusion of innovations theory to guide reporting of the study findings.

**Results:**

The study recruited 59 participants (35 male and 24 female), across the different data collection methods: round table discussion (16 participants), key informant interviews (10 participants), focus group discussions (7 participants), and stakeholder meetings (26 participants). We found a widespread lack of understanding and awareness of AI among both health workers and the general community. Among participants who shared their perspectives, 2 overarching themes emerged around the current and potential uses of AI innovations: filling gaps when skilled and experienced health personnel are not consistently available and targeting high-priority health activities or patients. Participants also emphasized several critical considerations. Building trust among health care providers, patients, and the broader community was seen as essential, alongside addressing language and cultural diversity, both of which require deliberate capacity strengthening. Ensuring equity and sustainability through cocreation strategies was equally stressed. Key concerns raised included ethics, cost, health literacy, and data biases.

**Conclusions:**

Our findings will inform the development of a continent-wide AI hub for MSRH, highlighting barriers and opportunities for improving health care access. We aim to support policymakers, researchers, and implementers in using AI to promote equitable maternal, sexual, and reproductive health care delivery across Africa.

## Introduction

The World Health Organization estimates that poor reproductive health accounts for up to 18% of the global burden of disease, and 32% of the total burden of disease for women of reproductive age [1]. In sub-Saharan Africa, death and disability resulting from reproductive health causes remain unacceptably high. The adult lifetime risk of maternal death has been estimated to be highest in Africa (1 in 55), while developed countries have been estimated to have the smallest lifetime risk (1 in 7410) [2]. The prevention and control of reproductive tract infections is another area of concern; for example, congenital syphilis is the second leading cause of preventable stillbirth globally, preceded only by malaria [3]. The global HIV response continues to be undermined by a multitude of complex, interrelated challenges that are aggravated by limited domestic resources, declining donor assistance, including the recent reorganization of US global health commitment, which threatens the sustainability of all critical health programs in the region. Vulnerable populations such as adolescents in Africa continue to be more susceptible to sexually transmitted infections, HIV, unwanted pregnancies, and unsafe abortions [4,5].

In the last 5 to 10 years, there has been an explosion in available health data in Africa due to improved infrastructure for electricity and internet as well as the widespread adoption of digital health technologies [4,6,7]. This provides an opportunity for data-driven strategies and innovations that previously did not exist. To increase technology-driven solutions, we must consider the human research and implementation capacity to enable this valuable resource to improve the health of the extremely diverse population.

Due to the rapidly emerging technology around AI, many health care workers and policymakers are not aware of the opportunities and limitations of these technologies. The use of AI in health in low- and middle-income countries is now emerging across sub-Saharan Africa. There is consensus in the literature that there is potential, along with risks, in using AI in expanding and extending health care access, by contributing to early disease detection and prevention, increasing diagnostic capability and drug development, disease surveillance, stock and health care management, as well as clinical decision-making [8].

The overall goal of this study was to explore the early experiences, perceptions of health workers, policymakers, and the general population, as well as AI researchers and implementers around the opportunities, risks, limitations, and best practices for responsible AI applications in maternal, sexual, and reproductive health (MSRH) in sub-Saharan Africa.

## Methods

### Study Overview

**Figure 1.**
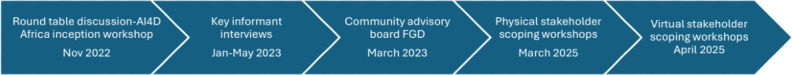
Timeline of qualitative data collection activities, sub-Saharan Africa, 2022‐2025. AI4D: Artificial Intelligence for Development; FGD: focus group discussion.

We conducted an exploratory qualitative study involving key informant interviews, focus groups, and stakeholder meetings to examine perceptions of health workers, policymakers, AI researchers and implementers, as well as the general population and community advisory board (CAB) members (Figure 1). We delved into the opportunities, risks, limitations, and best practices for responsible AI in MSRH in sub-Saharan Africa.

The study was conducted within the activities of The Hub for Artificial Intelligence in Maternal, Sexual and Reproductive Health (HASH) in sub-Saharan Africa. HASH was formed in 2021 by a multidisciplinary consortium of the Infectious Diseases Institute, the Makerere University College of Computing and Information Science, Sunbird AI. HASH is funded by the International Development Research Centre and the Swedish International Development Cooperation Agency, as part of the Artificial Intelligence for Development in Africa Program (AI4D Africa) and the Global South AI for Global Health (AI4GH) initiative. The Consortium aims to advance MSRH and rights while strengthening health systems in SSA through the responsible development and deployment of AI innovations.

Using the HASH platform, we purposively sampled thought leaders in AI in MSRH for targeted focus group discussions and in-depth interviews. (Multimedia Appendix 1) Additionally, we collected data from routine stakeholder engagement consultations (called scoping workshops) to inform the strategy and implementation of HASH. Through this varied methodological approach to purposive sampling and data collection, we have a wide-ranging view of knowledge, experience, and attitudes regarding responsible AI in MSRH in sub-Saharan Africa.

### Theoretical Framework

Diffusion of innovations theory (DOI), developed by Everett Rogers, explains how new technologies and ideas can spread through a social system in a predictable way, with individuals adopting innovations at varying rates based on risk tolerance, social influence, and access to information. The theory emphasizes key factors such as communication channels, perceived attributes of the innovation, and observability; the ability to witness community members benefiting from the innovation in everyday life, as critical drivers of adoption [9-11]. DOI provides this scaffolding to understand how innovations are adopted, highlighting knowledge, attitudes, barriers, and recommendations for the adoption of AI in MSRH in the future within numerous environments in sub-Saharan Africa. We inspect our data through a DOI lens and loosely describe the narrative to explore factors influencing stakeholder engagement in AI applications.

### Recruitment, Sampling, and Data Collection Procedures

#### Round Table Discussion (RTD)

A round table discussion (RTD) was held during the in-person AI for Development Inception Workshop in Senegal in November 2022. All participants were invited, and all willing workshop attendees, researchers, and implementers of AI in sub-Saharan Africa self-selected by attending a designated session included on the official agenda (convenience sampling). The participants were consulted on their perspectives regarding the opportunities, risks, limitations, and best practices of AI in MSRH. They received a detailed explanation of the purpose and procedures of the RTD and signed an attendance form approved by the Research Ethics Committee (REC), indicating their consent to participate. A discussion guide for the RTD was managed by a moderator, with a note-taker to record audio and take additional notes during the discussion. The process lasted for 50 minutes, was interactive, and the RTD was conducted face-to-face in English.

#### Participant Selection; Key Informant Interviews (KII)

As part of HASH activities in 2021, we conducted an online survey involving 107 MSRH experts and AI researchers from 25 countries across Africa. We selected our qualitative participants from the 107 in this previous survey; thus, including individuals with evidence of expert knowledge, including but not limited to: actively working on AI projects in health care, working in MSRH, familiar with the concepts and methods of AI, based in anglophone or francophone sub-Saharan Africa. During the online survey, all respondents were asked if they could be contacted for further one-on-one interviews. A total of 21 eligible survey respondents were then shortlisted to participate in the key informant interviews (KIIs) by 2 investigators (EO, RK) using purposive sampling by criterion. The criterion used to determine eligibility for KII was the level of expertise and experience in either MSRH or AI as reported in results from the HASH online survey [12]. Respondents were contacted via email or telephone, and appointments were made.

Eleven of the 21 eligible respondents were screened out due to nonresponse or failure to comply with interview scheduling. Before the interview, the participant received a consent form via email. During the scheduled interview time, informed consent was administered by the interviewer, and the participant was asked to voluntarily sign the consent form with an electronic signature and email it to the interviewer before the interview began.

#### Participant Selection; Focus Group Discussion (FGD)

Participants were invited to the focus group discussion (FGD) if they were a member of the Academy for Health Innovations CAB at the Infectious Diseases Institute in Uganda. The 9-member CAB is composed of Ugandan nationals, namely ministry of health officials, a youth representative, a health worker from a lower health facility, 2 religious leaders (Muslim; Catholic), a local council leader, a microfinance administrator, and a civil society organizational representative. The CAB’s main role is to advise and facilitate dialogue between the community and the research team. An appointment was made for the FGD with all the CAB members either face-to-face or online through an email or phone. The discussion was conducted in March 2023, with 7 participants (4 men and 3 women), 5 physical and 2 virtual. All respondents were consented in the same manner as for the interviews.

#### Data Collection Interviews and FGD

A standardized semistructured guide was developed for FGD and KII. We aimed to explore the perceptions of the stakeholders on the future of AI in health care in Africa, including perceived benefits and limitations. Exploration of the role in MSRH was based upon the priority areas identified in the HASH quantitative survey (posted on HASH website). Each interview was held with 2 Ugandan social scientists; one to guide the discussion and one to take notes. The process was interactive and took between 30 and 60 minutes depending on experience with AI. All KIIs and FGD were conducted in English as we did not have anyone preferring French or any other language when they were asked.

The interviews were conducted either face-to-face or by Zoom (noted in Table 1) with video on for the participant, moderator, and note-taker throughout the interview to allow for recognition of nonverbal cues. The FGD was face-to-face, with 2 participants online. All sessions and recordings were kept in a secured file in an online repository with access restricted only to the study team members. The FGD started off with a 45-minute presentation that explained AI, its application in everyday life and in health care, as well as the principles of responsible AI and how HASH is contributing to the space. This presentation ensured shared understanding and set the scene for rich discussion.

**Table 1.** Qualitative study data collection methods, format, gender and numbers of participants, sub-Saharan Africa, 2022‐2025.

Data collection method	Format	Gender and number of participants			Participant characteristics
		Men	Women	Total	
Round table discussion - AI4D^a^ Africa Inception Workshop	In-person	10	6	16	2 Professors 4 Researchers 1 Civil society advocate 1 Data scientist 1 Program manager 3 Principal investigators 1 Cofounder 1 Partnership coordinator 1 Computer scientist 1 Legal officer
Key informant interviews	Virtual and in-person	7	3	10	1 NGO^b^ founder 2 Senior managers 2 Managers 1 Professor 1 NGO staff 1 Data officer
Community advisory board focus group discussion	Virtual and in-person	3	4	7	1 Senior nurse 1 Lab technologist 1 Community leader 1 Retired teacher 1 Civil servant 2 NGO Heads
Physical stakeholder scoping workshops	In-person	5	6	11	1 AI innovator 2 Civil society advocates 1 Obstetrician gynecologist 1 Policymakers 4 Health entrepreneurs 2 Health educators
Virtual stakeholder scoping workshops	Virtual	10	5	15	2 Religious leaders 1 Foundation staff 2 Young people 1 Policymakers 2 Health entrepreneurs 1 Venture capitalists 3 Health educators 1 Civil society advocate 2 Innovators

a AI4D: AI for development

b NGO: non government orgnaization

#### Scoping Workshops (n*=3*)

Three multistakeholder workshops with participants across sub-Saharan Africa convened diverse voices including health care providers, AI innovators, venture capitalists, health educators, policymakers, young people (15‐24 years), health entrepreneurs, and religious leaders to design a path forward regarding AI innovations and MSRH in sub-Saharan Africa. Participants were identified through stakeholder mapping using criterion purposive selection aiming to identify representatives across the above key categories who could clearly express views on our research question. The 8 categories (health care providers, AI innovators, venture capitalists, health educators, policymakers, young people 15‐24 years old, health entrepreneurs, and religious leaders) of stakeholders were listed, and a committee brainstormed all potential stakeholders per category. All potentials listed were invited to attend. At the invitation stage and at the beginning of the workshops, participants received a full explanation of all aspects pertaining to the study and documented their willingness to participate on the same REC-approved registration form as for the RTD. The workshops included presentations on responsible AI, on the HASH project, and on scaling impact in developing countries to facilitate a level playing field for fruitful discussion. The sessions were designed to capture diverse stakeholder perspectives on the role of AI in MSRH. One full-day physical workshop in Uganda, with Ugandan participants, and 2 half-day virtual workshops with participants from around the globe were held in March and April 2025. Each workshop convened participants from across the AI in MSRH ecosystem to foster rich, multidisciplinary dialogue in the sub-Saharan contexts. Four social scientists were present as participant observers to critically observe and document the full discussions, reactions, and content of each session.

For the scoping workshops, the insights from stakeholders were structured around five guiding questions: (1) Why do we need AI in maternal, sexual, and reproductive health? (2) What strategies enable AI to meet the needs of all stakeholders in MSRH? (3) What are the concerns and anticipated challenges of AI in MSRH? (4) What are the overall implications and way forward? (5) How do we make AI a mainstream tool for health in your setting? All discussions were held in English and lasted for 2 hours and 30 minutes for virtual workshops and 4 hours for physical workshops (divided into 2 breakout sessions). All workshops were audio recorded.

### Data Analysis

RTD, FGD, and KII audio recordings were transcribed verbatim. All of our data were analyzed using the framework analysis approach, while the scoping workshop data additionally layered the guiding questions mentioned above [13-15]. Framework analysis produces highly structured outputs of summarized data and is particularly useful for large quantities of data. Framework analysis is commonly used for the thematic analysis of semi-structured transcripts as reflexivity, rigor, and quality are integral and critical components of the approach. We implemented the 5 stages of framework analysis systematically with 3 coding team members (RK, EO, CA). In the familiarization stage, all three team members independently read and reread the full transcript, making preliminary notes on recurring ideas, patterns, contradictions, and early impressions. These notes informed the development of an initial thematic framework that combined deductive codes derived from the research questions with inductive codes emerging from participants’ accounts. The team met to compare preliminary interpretations, refine code definitions, merge overlapping codes, separate codes that captured distinctive meanings, discard codes not sufficiently grounded in the data, and agreed on the final coding framework. When differences in interpretation arose, the team returned to the relevant transcript excerpts, considered the broader interview context, and compared similar excerpts across transcripts and participant groups before reaching consensus.

In the charting stage, coded data were lifted from their original context and reorganized into thematic charts, allowing us to compare patterns and construct summaries for each theme across all participants. Finally, in the mapping and interpretation stage, we examined the charts to examine how codes clustered into categories, how categories related to one another, and how they explained the participants’ experiences. Emerging themes were refined iteratively through team discussion and checked against the original data to ensure the final themes represented the range of the data and reached a shared analytic understanding. To ensure credibility and confirmability, we presented preliminary results to check with the members to verify accuracy on 3 occasions (Table 2). For dependability, we emphasized documenting our research process thoroughly through Standard Operating Procedures (SOPs), workshop concept notes, and activity reports. To enhance transferability, we took time to reflect on what criteria to use in our purposive sampling approach to ensure that the findings can be applied to similar populations, contexts, and settings. In the last stage of the analysis, we loosely mapped our themes in relation to the DOI themes as presented below in the findings.

**Table 2.** Dissemination events where preliminary study findings were discussed, sub-Saharan Africa, 2023‐2025.

Event	Format	Dates
Inaugural AfricaAI Conference, Kigali, Rwanda	Research paper session	June 2023
AI4GH^a^ meeting, Nairobi	Presentation	November 2023
Technology and Innovation unit UK^b^ Foreign, Commonwealth Development Office	Presentation	January 2025

a AI4GH: artificial intelligence for global health

b UK: United Kingdom

### Reflexivity and Positionality

Our research team comprised both African and non-African researchers of a large age range, whose diverse positionalities are shaped by nationality, gender, and varying proximity to the communities and health systems studied. (Multimedia Appendix 2) This diversity inevitably influenced our interpretation of our data collection process and analysis on AI’s role in MSRH in these settings. To address this, each team member documented their positionality during data collection, and these reflections were revisited collectively during analysis to acknowledge and interrogate divergent interpretations, ensuring that reflexivity was an active and ongoing analytic practice rather than a procedural formality. One of our discussion points in both the data collection and the analytic practice was around differences in age and exposure to AI amongst ourselves and our participants in the different data collection exercises. Upon reflection, we discovered that our participants were similar in age, education, and exposure to ourselves. These discussions enabled a shift in later data collection to ensure younger participants were included. With regard to analysis, reflecting on our own access to AI and assumptions that younger people may have greater facility with AI, in analysis discussions we foregrounded the issues of age and exposure with respect to our own attitudes, experiences, and beliefs.

We combined RTD, scoping workshop, KII, and FGD data. The preliminary findings were presented for feedback on 3 occasions: inaugural AfricaAI conference 2023 in Rwanda; AI4GH meeting in Nairobi in November 2023; and to the Technology and Innovation unit at the UK Foreign, Commonwealth Development Office in January 2025 (Table 2)

### Ethical Considerations

Ethical approval was obtained from the Infectious Diseases Institute Research Ethics Committee (IDIREC REF 011/2022) and the Uganda National Council for Science and Technology (HS2356ES) and covered multi-country participation. The study was conducted in accordance with the protocol, GCP guidelines, the Declaration of Helsinki, and all applicable local regulatory requirements and laws. All research team members had verified GCP certification.

Data were anonymized, and no personal identification was collected. Participants at the RTD did not receive any compensation. FGD participants received refreshments and US $11 to US $28 as transport reimbursement depending on the distance. KII participants received US $35. Physical scoping workshop participants received refreshments and US $87 as compensation. Virtual scoping workshop participants received US $87 as compensation.

## Results

### Overview

We include 59 total participants as described in Table 1 below from Uganda, Nigeria, Kenya, Tanzania, Ghana, Zambia, and Senegal, with similar numbers of men and women. We included 11 participants in the first in-person scoping workshop in Uganda and 15 participants in the 2 virtual scoping workshops where we had participants from across the continent (Table 3). The FGD included 7 participants, and the RTD had 16 participants. The KIIs had 10 participants, but due to a technical issue with the recording, one KII was repeated to capture the last half of the interview.

**Table 3.** Country representation of participants in virtual scoping workshops qualitative study, sub-Saharan Africa, 2022‐2025*.*

Characteristic and category	Participants, n (%)
Gender	
Men	10 (66.7)
Women	5 (33.3)
Country	
Uganda	6 (33.3)
Kenya	2 (13.3)
Nigeria	3 (13.3)
Zambia	2 (13.3)
Ghana	1 (6.7)
Senegal	1 (6.7)

### Diffusion of Innovations Theory Findings

**Figure 2.**
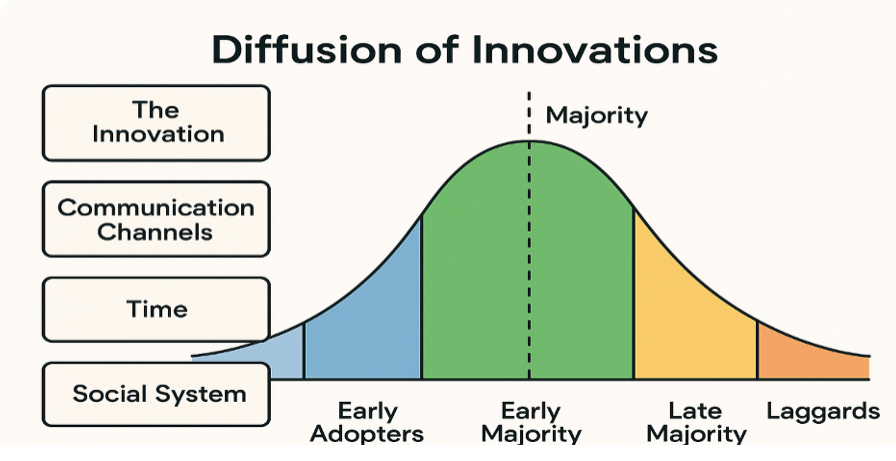
Diffusion of innovations theory applied to qualitative study in sub-Saharan Africa, 2022‐2025.

We describe our findings based on the 4 main elements in the DOI theory: the innovation, communication channels, time, and the social system (Figure 2). For innovation in this case, we consider a cluster of potential or existing AI innovations; for the time element, we look specifically at the implementation or actualization of AI or MSRH projects.

#### The Innovation

##### Current and Suggested Uses of AI

When asked about participants’ current and suggested uses of AI in MSRH, helping to target or add specificity to high-priority health activities or patients was an overarching theme.

Some participants mentioned how useful AI has been in tracking logistics, such as tracking supply chain products and triggering action specifically for security purposes and when or if the terrain or environment was challenging. Other participants stated how powerful chatbots are in personalizing care by answering health-related questions. Additionally, participants reported that AI could be used to rapidly triage numerous queries from health service users to prioritize questions that required urgent responses. Participants highlighted that these questions and answers could then be used to develop datasets such as ultrasound imaging.

*We try to develop predictive models to deliver care more efficiently and to deliver the care to the women who need it. So, there are some services we deliver as a blanket care to everybody but if we identify certain risk factors or certain special needs, then we think, predictive modeling through machine learning or through AI, can help us identify women or children who need specialized care, then we could deliver that care to only these clients which makes the service delivery more targeted because we do not need to provide these generalized messages. We would only provide messages that are relevant to that client*.

[KII, male, NGO manager TZ]

In the quote above, participants highlighted the importance of AI’s ability to target specific services and information that is tailored to patient needs, rather than providing the same general service and information to everyone. This could help in improving patient-centered care and in resource allocation.

*with the Q and A functionality; what we used to do is answer questions in the order in which they arrived. If… we got…. in a day [more than] 90 questions that were not urgent, and number 91 was something that needed immediate attention, we would have to answer the questions 1 to 90 before we even realize that 91 was something that needed immediate attention.*

[KII, male, NGO, Kenya]

Many participants in the scoping workshops, as well as in the interviews and FGD, emphasized that they felt that the use of AI was not to replace current roles or individuals but to improve efficiency and enhance numerous medical services. Those who are working in AI and those that expressed positive attitudes for using AI might be considered early adopters of AI.

##### Benefits of AI

General benefits that participants stressed included using AI to fill gaps, particularly when skilled and experienced health personnel were not continuously available, especially in rural and hard-to-reach areas. It was mentioned that AI can, in principle, eliminate human error and corruption, and therefore should support equity as well as reducing time and resources spent on care. Participants felt that with these advantages, we could save money and reduce workload while increasing coverage and quality of health services. Many felt that AI can increase accurate health information and improve collaboration between providers.

*AI is competing with the best human skills and so it is able to get you more accurate support in terms of diagnosis and is able to do it more quickly.. . . . , in terms of being able to support with diagnosis in a timely manner which high accuracy, AI is quite instrumental, and it is low cost especially if it can come as open source. Bias too is eliminated. . . not totally eliminated. Biases are always there but when we give a lot of our data to AI algorithms, let’s say Nigeria’s or South Africa’s data, they tend to, over time, be able to predict more accurately our needs regarding to patients’ diagnosis*.

[KII, male, professor, Nigeria]

When asked about which populations may benefit the most from an increase in the use of AI, respondents highlighted high literacy populations because AI innovations often use technology through text. Additionally, rural populations who may have limited access to conventional medical services and adolescents who adopt technology quickly stand to benefit highly, though one participant from Nigeria stated that all populations will benefit from AI.

*fishermen, as they are so migratory because today they are here, tomorrow they are there.*

[CAB FGD, male community leader, Uganda]

Content areas that KII participants reported benefiting most from AI included maternal and newborn health as well as sexual and reproductive health. Within MSRH, specific areas that were stressed revolved around monitoring of health conditions including pregnancy, blood sugar for gestational diabetes, postnatal monitoring, and pregnancy complications.

*There are times when the baby is having a temperature. It may not necessarily need to go to hospital. . . .It (AI) can give you some form of first aid that can do (in the moment*).

[KII, female, manager For-Profit Organization, Nigeria]

*I think one of the things that AI can help (with) at community level is to develop software that can tell this mother about the dangers of the baby.*

[CAB FGD male, civil servant, Uganda]

Some growth areas that participants noted for further development would be in using images to build databases.

*What I discovered about that ultrasound is… I saw that they take pictures and if that picture is well scanned, you can create a whole dataset*.

[KII, man, NGO staff, Uganda]

### Communication Channels

Communications channels are a key element in relation to the adoption of new innovations. An overarching, though not a surprising result, was the lack of understanding and awareness of AI among health workers and the general community. From health workers worried about job security to patients new to digital tools, the importance of user education was a key finding.

Ensuring AI meets people’s needs also requires capacity building and education. A scoping workshop speaker, who works closely with frontline health workers, pointed out that many providers and patients currently have limited understanding of AI. To prevent AI tools from sitting unused or being misused, investment in training is vital.

*Even the best AI tool is useless if health workers don’t know how to use it or don’t trust it*.

[Scoping workshop]

Addressing fear around trust in AI would necessitate building confidence through education and transparent processes. Encouraging partnerships and enforcing collaboration through donor requirements may improve outcomes. One strategy discussed in a scoping workshop was to incorporate AI literacy into medical and nursing education, as well as offering continuous training for existing staff where AI systems would be introduced.

Once health workers decide to adopt AI, then clear user guidance is necessary to mitigate harm from inaccurate queries or incomplete data. Health workers should learn not just how to operate AI-driven devices or apps, but also how to interpret AI outputs critically and integrate them with clinical judgment. One participant noted the significance of training on

*how to prompt AI…so if you get incorrect information, you are going to go with that wrong information.*

[Scoping workshop]

Likewise, community health educators could help familiarize the public with new AI tools and services (for example, teaching expectant mothers how to interact with an AI-driven messaging service that sends them prenatal care advice). The goal of these capacity-building efforts is to ensure that AI becomes a help rather than a hindrance in the workflow, and that communities feel empowered rather than intimidated by new technology. Other suggestions in the FGD included educating the general public about what AI is and what AI is not.

The CAB members also suggested using AI in an integration process for both traditional and biomedical health care systems. Traditional birth attendants (TBAs) are highly valued in some communities in sub-Saharan Africa and may be an important communication channel for AI in MSRH.

*I think the best way is to involve the TBAs, first, to educate them and show them what they can do and what they cannot do. That can give them contacts which they can refer to ... [their strength] is ... about the customer care that they show*

[CAB FGD, male civil servant, Uganda]

Ensuring a cultural fit, especially with respect to linguistic barriers, was a key point highlighted in the scoping workshops. Recognizing that MSRH is deeply intertwined with cultural norms and sensitivities is paramount. AI systems and all potential solutions, particularly in diverse contexts, like in Africa where open discussions about sex are often taboo, must undergo thorough cultural reviews. The communication style, information delivery, and even the platform’s interface should be sensitive to local customs and communication patterns. Creating a sense of trust and comfort is essential, as demonstrated by the strategy of designing chatbots with a friendly and approachable persona.

Many participants cited rural populations with limited English proficiency as an example for ensuring localized AI solutions. One scoping workshop speaker asked,

*Is it possible for me to deposit my Ateso [local language in Uganda] somewhere so that it can be utilized…*?

[Scoping workshop]

This underscores the urgent need for AI-driven tools that incorporate local languages and dialects, ensuring that critical MSRH information is accessible to all.

Cocreation and inclusive design were mentioned as key strategies to tackling the language and culture concern. Several contributors advocated for a cocreation process involving health workers, community members, and technology developers. “It’s important to have a co-creation process in the way that AI is developed,*”* (scoping workshop) as one attendee mentioned. As noted in a scoping workshop, this cultural tailoring is vital when implementing AI in Africa, where stigma around sexual health influences care-seeking behaviors.

*…ensuring it’s relatable to the daily experiences of these (specific) communities [is a key to successful uptake].*

[Scoping workshop]

Such collaboration ensures user-friendly designs that accurately reflect community knowledge, practices, and ethical priorities. Additionally, when communities feel a sense of ownership over a tool, they are more likely to trust it and use it.

In addition to the language and cultural concerns, several scoping workshop participants also touched on the importance of interdisciplinary collaboration to breaking down silos between technology developers and health care practitioners, social scientists, and the intended beneficiaries of the technology. One scoping workshop participant emphasized:

*We learned that when engineers sit with midwives and doctors, they come up with much more practical solutions. Neither can do it alone — it has to be a joint effort*.

Through collaboration, it was mentioned that AI solutions are more likely to address real-world problems in a feasible way. One illustrative example from the discussion in a scoping workshop was a pilot project mentioned by one scoping workshop participant, where an AI tool for predicting postpartum hemorrhage risk was developed with direct input from obstetricians: the clinicians specified what risk factors they saw as red flags, and the engineers used those insights to train a model that aligned with clinical intuition. Such collaborative models help ensure the resulting AI is not a “black box” but something clinicians feel connected to. Participants in scoping workshops, FGDs, and KIIs were all asked about priorities for research and development. Some suggestions highlighted the importance of communication channels. CAB members signaled the area of how to incorporate AI in building awareness for high-risk health issues at the community level to prevent emergency situations. Another CAB member suggested not to focus only on the mother, but to include other family members such as the male partner in pregnancy-related awareness raising.

### Time, Implementation and Actualization in AI Solutions

#### Challenges That Impact Time From Innovation to Diffusion

Participants’ concerns revolved around 6 main areas including: ethics and regulation, cost, specifically internet accessibility costs and sustainability, trust in new innovations, access including population literacy, data and bias, as well as cultural and religious barriers. One participant mentioned that like current use of Google, there could be an increased risk.

*In situations where you are supposed to go and see your doctor, you are relying on what your chatbot or your application is telling you. You misuse it. ... It may lead to abuse of information, abuse of access to information you are supposed to use positively but people tend to abuse it*.

[KI, female manager, For-Profit Organization, Nigeria]

#### Regulation and Ethical Concerns

Marginalized populations, including women in patriarchal societies, could face barriers to benefiting from AI. Confidentiality breaches were mentioned as a concern with use of AI. Regulatory frameworks have not caught up to the pace of innovations in many sub-Saharan Africa countries. AI expert participants described some of the complexities ranging from data literacy and technology access bottlenecks.

#### Data and Bias Issues

In addition, both community and expert participants in all of our activities highlighted that AI models can face limited data sources in low-research languages and regions, compounded by biases in existing datasets. Participants felt that this can lead to inequitable outcomes, especially in marginalized communities.

*Access to health data is not straightforward because of the sensitivity of the information; privacy is very important. Stakeholders do not always understand the importance of AI tools and hence are skeptical about having health data shared with developers. Lack of a central repository for data resulting in segmented data. There is need for data, otherwise AI cannot exist. Data acquisition is a challenge. Data entrants may not fully understand the concept of unique identifiers and may choose to use traceable data eg, phone numbers as a unique ID. . Medical workers and data entrants may not understand how to encrypt data; lack of knowledge on data encryption may limit data sharing where it would otherwise be possible. We must be careful what data we train AI on because what it is fed is what it gives. Not all data is good for AI. In many cases the data being used was not initially collected for AI use, so there may be a lot of missing data elements or assumptions. Data acquisition takes a long time and causes a lot of frustration to developers, especially because it involves a lot of stakeholder engagement.*

[KII Uganda]

#### Cost, Including Connectivity and Infrastructure

All groups of participants noted that limited internet access and inconsistent electricity in low-income regions could lead to either low uptake or low capacity to sustain these projects.

#### Trust and Literacy

It is common across the literature that initiation of new innovations often instills hesitancy. This was found in our data as well that adopting AI instills some fear of change, concerns about job loss, and low education levels, specifically literacy levels and more specifically in areas and populations with low data literacy among some population groups including community health workers. Some participants mentioned concern that their community would not know how to use AI correctly, thus could endanger their health instead of benefiting. A lack of collaboration and data sharing between stakeholders hinders progress.

#### Cultural and Religious Barriers

Some communities resist technological innovations due to religious beliefs or cultural norms. Additionally, the belief in the importance of human-to-human connection and the risk of losing that with increased use of AI.

*someone can misinterpret the guidelines or the information that AI is using to maybe give treatment or diagnosis and come up with a decision that will affect the patient in due course . . . certain people believe that if I go to the health center and I don’t find Stella, I might not be treated well, I would rather go to someone I know is not even a health person but just talking to her or him might cure me.*

[CAB FGD, female retired teacher, Uganda]

### Social System

Generally, within the DOI Theory, one considers the social system as including social norms, cultural values, social networks, political factors like government policies, ethics, regulations, and infrastructure.

Our data suggested that policymakers need to think ahead for sustainability to ensure capacity building, due to the complexity and layers involved in sustaining these systems. There were multiple examples highlighted of knowledge, experience, and attitude gaps that will hinder sustainability.

(*for sustainability) we need to find ways to engage the opinion leaders, ..., we need to explore the use of experts (to educate opinion leaders*).

[CAB FGD, male Community Leader, Uganda]

*we need to have a systems thinking approach however hard it is.*

[CAB FGD, male civil servant, Uganda]

All groups of participants stressed that tailored, human-centered, engagement and inclusive strategies would be necessary to overcome barriers. Addressing challenges requires a holistic approach, combining technical solutions that work with societal, educational, and policy interventions to strengthen the diffusion of any of the AI maternal health innovations within the social system.

A key point highlighted in a scoping workshop was the strategy of continuous evaluation and feedback. One speaker noted that implementing AI in MSRH should be an iterative process:

*We should deploy AI carefully and study its impact. Are mothers actually healthier? Are providers happier? We need to gather feedback and be ready to improve the tools*.

In practice, this would imply using traditional clinical evidence methods such as randomized controlled trials for efficacy compared to humans alone, as well as monitoring outcomes once an AI system is rolled out (for example, tracking if an AI triage system actually reduced waiting times at clinics or improved maternal mortality rates in an area). Getting feedback from users would allow developers and health authorities to refine the system or even decide to scale it up or down. This approach would, if working well, ensure that AI remains aligned with the people’s needs over time, adapting to new information or changing circumstances in close to real time.

With regards to the ethical concerns, participants mentioned strengthening ethical protocols by ensuring data protection and combating institutional biases. It was emphasized that it is key to ensure that there is always a human in the loop; this is important to the general population in addition to the specifics for MSRH.

To alleviate infrastructural issues such as the cost of or lack of internet, participants suggested that future models would necessitate AI models that can function offline and remain efficient under resource constraints. In the FGD, some reflections on sustainability revolved around how use of cultural opinion leaders, scientific experts, and other stakeholders enable a longer-term vision and can contextualize AI use for MSRH in sub-Saharan Africa.

Participants in the scoping workshop cautioned that using AI cannot instantly remove all the challenges in our MSRH services; it will only provide one piece of the complex puzzle.

We need AI precisely because we need to do better for mothers and young people. But AI is not a silver bullet for all our health system problems, the participant cautioned.

[Scoping workshop]

## Discussion

Across the participant groups and data collection methods, we found a high level of diversity in the experiences and perceptions of AI in MSRH in sub-Saharan Africa. In high-income countries, AI in MSRH is predicted to touch all areas of health care from disease prediction to health promotion in the very near future [8]. Our data clearly highlighted that many stakeholders felt that AI could benefit MSRH because it offers assistance through powerful tools to improve outcomes and equity. Our findings showed that participants could envision potential value of AI innovations as a support tool to strengthen MSRH health care delivery including: improving stock management and triage, increasing MSRH awareness, enhancing diagnosis, guiding treatments, and enabling personalized care. However, our results suggest that this same level of confidence in AI for MSRH among early adopters has not been reached to allow for a wide diffusion and for the potential of AI in MSRH among the diversity of African stakeholders.

A global systematic review of health care professional experience by Ayorinde et al identified health care professionals’ understanding of AI applications, level of trust and confidence in AI tools, and judging the value added by AI as main areas raised in their study [16]. In Africa, there have been several quantitative interview studies of health worker perceptions including trainee radiologists [17,18]. A comprehensive mixed methods study by Asiedu with health workers and the general public across Africa revealed more positive attitudes towards AI from the general public compared to more cautious views from health workers who identified trust, ethics, and systemic barriers to integration [18]. For MSRH-specific work, a review of midwife perceptions revealed 8 studies highlighting potential for improved quality of care, particularly in perinatal and neonatal settings, with barriers to integration of ethical concerns and hesitation among midwives, due to low levels of digital health literacy.

Participants in our study were steadfast in emphasizing the core principle that AI should augment, not replace human health care. This finding was present across AI professionals and the general public. It also mirrors findings in high-income settings (HICs) as identified by El Arab et al in a systematic review of 37 studies [9]. This suggests that the perceived value of the “human touch” in health care cuts across contexts. As other studies have highlighted lack of awareness of health workers about the opportunities of health AI; health workers are thirsty for knowledge about how they can use AI, and how to make sure it is used safely and appropriately. The finding from this study that cocreating AI tools with health workers, traditional healers, and communities, rather than developing tools for them, helps to ensure that the tools are both safe and equitably beneficial.

Our data reinforces previous experience with health technology adoption, that it must go hand-in-hand with strengthening health care infrastructure and workforce to effectively use the innovations. The consensus across our data, as well as others, cautions that AI must be implemented thoughtfully and carefully alongside improvements in capacity, regulation, and infrastructure [8,19]. Rigorous evaluation of new AI tools pre- and during implementation was highlighted by participants as an important process that would support trust in the tools. Our findings confirm this as a significant disparity between the AI-related capacities of sub-Saharan Africa and HICs. In HICs, internet access is as high as 93% in some countries, electricity is reliable, there is a large concentration of AI professionals, and discussions on the available datasets usually surround biases and representation. In sub-Saharan Africa, however, internet coverage is commonly below 27%, electricity shortages are common, and locally representative datasets are often completely absent, all of which are factors that negatively impact the development of local AI talent. These differences create a disparity in global potential to equitably leverage the benefits of AI [10].

Ensuring adequate and nonbiased data was a concern that was less prevalent in health worker–facing studies; this may be due to the inclusion of AI experts in this study [17,20,21]. This is particularly important in MSRH, as women can be more frequently missing in research data, formal medical records, and other socio-economic datasets. Notably in our study and in others were the challenges related to ethics and regulation [22].

Widespread adoption of AI in sub-Saharan Africa will require agility and strong consideration of differences between sub-Saharan Africa and non-sub-Saharan Africa contexts. For example, sub-Saharan Africa is unique in that there are over 2000 languages [11]. This remarkable diversity presents a significant inclusion challenge where in HICs this challenge is commonly overcome by only considering 3 languages - English, French, and Spanish [12]. AI tools developed in these 3 languages will not suffice in ensuring global equitable AI access and will require adaptation in sub-Saharan Africa. Additional adaptation will be required in terms of algorithmic validation and retraining on African data to ensure data representation, diversity, and to mitigate algorithmic bias [13]. A key example of this is the limitation of AI in detecting skin cancer on dark skin [14]. A successful attempt at this is the crowdsourcing by Oseku et al [23] of an African dataset of question-and-answer pairs on sexually transmitted infections that captures African concerns and terminology around sexual health. AI will also have to be deployed within the existing infrastructure limitations, necessitating deployments that are offline or use low bandwidth [13]. Finally, inadequate AI regulatory frameworks in sub-Saharan Africa risk reinforcing power asymmetry and deploying what has been built elsewhere limits the capacity for sub-Saharan Africa to detect biases, dangers, and to protect patient rights. Therefore, positive advancements such as the continental artificial intelligence strategy by the African Union will require deliberate adoption and operationalization to turn them into effective governance and implementation [24].

AI innovations have been highlighted as a potential to revolutionize maternal and other health care in Africa, yet large-scale studies are limited across the continent [8,25]. Our study found that subject matter experts believe there are diverse AI innovations that could improve, widen the reach, and increase equity in MSRH, yet their diffusion depends on a number of factors related to all 4 key pillars of the theory; the innovation itself that is built on complex and costly infrastructure, the communication channels that depend on accessibility to technology, the speed with which access is available for new innovative projects, and the social system comprised of cultural and language challenges. We referred to the DOI theory in our analysis of findings to help frame our results. We found that diffusion of AI innovations has been hampered by awareness in the general public of specific AI innovations, complexity of many of the innovations, trialability, observability, and in the realm of ethics and regulations [26].

We have some limitations to our study. First, though we were prepared to have Anglophone and Francophone participants, through the online survey being bilingual and the option for translation being available for the KIIs, we did not have any participants requesting to speak French. An additional potential weakness of this study is that there was a large gap in time between KII-FGD and the scoping workshop (Figure 1), and we were initially expecting results to be very different and require separate reporting; however, during analysis, the themes were more similar than expected, so the data were combined. Finally, the study did not capture participants’ ages; therefore, the extent of age-range representation could not be determined.

The strengths of this study are the triangulation of 3 different settings, key informant interviews with international African AI and MSRH experts, FGD with a CAB, and observations at a scoping workshop (Table 1). This allows for a wide cross-sectional view of different perceptions of AI.

Overall, the study contributes to the growing body of evidence that African stakeholders – spanning AI experts, health workers, policymakers, and communities – all recognize that AI has great potential to tackle the urgent MSRH challenges that disproportionately affect women and adolescent girls, to improve quality and efficiency of MSRH health care delivery in Africa, and to widen the reach of health services. However, substantial conditions must be met before this potential can be equitably realized.

For the AI innovations to be disseminated equitably, an increase in awareness of the characteristics of AI innovations, their layered complexities, capacity to understand and use them fairly, and improving infrastructure will all be critical. Localization of algorithms and datasets, prioritization of offline AI capabilities, cocreation of AI tools with communities, health workers, and technologists, and rigorous regulation and evaluation may help to create safe, trustworthy, and easily adopted AI tools for MSRH.

## Supplementary material

10.2196/84378 Multimedia Appendix 1 Ranking of top research and development in MSRH according to potential and viability of AI as a solution, 2021, sub-Saharan Africa.

10.2196/84378 Multimedia Appendix 2 Personal Characteristics of the Research Team.

10.2196/84378 Checklist 1 COREQ checklist.
