# Supplementary material for: Exploring Perceptions of Leveraging AI to Improve Outcomes in Maternal, Sexual, and Reproductive Health in Sub-Saharan Africa: Exploratory Qualitative Study
Source: J Med Internet Res. 2026 Aug 13;28:e84378. doi: 10.2196/84378 (PMC13472523; doi:10.2196/84378)
Supplement: Supplementary file 2 [file jmir-v28-e84378-s001.docx]

***Ranking of top research and development in MSRH according to potential and viability of AI as a solution, 2021, sub-Saharan Africa***

| **THEME** | **PRIORITY AREA** **TO IMPROVE HEALTH OUTCOMES** |
| --- | --- |
| **MATERNAL HEALTH** | 1. Identification of high-risk conditions in mothers and newborns 2. Attracting and retaining skilled birth attendants in remote areas 3. Maternal mortality 4. Improving uptake of proven existing interventions 5. Algorithms and point of care diagnostic tests for sepsis in the face of emergencies and epidemics 6. Implementation research of interventions that improve quality of care during labour 7. The impact of training on skills of health workers handling obstetric emergencies 8. Improving access to emergency transport in hard-to-reach communities 9. Improved maternal and new-born emergency care |
| **ADOLESCENT SEXUAL REPRODUCTIVE HEALTH** | 1. Unmet need for contraception 2. Inform how health services can be designed to effectively meet adolescents' health needs. 3. Development of prevention technologies besides condoms 4. Gender gaps in sexual health 5. Examining health related behaviour (physical activity, diet) 6. Examining health seeking behaviour 7. Prevalence/incidence of different health outcomes (STIs, pregnancy) 8. Access to comprehensive SRH services 9. Evaluation of effectiveness of health service delivery models 10. Understanding needs of vulnerable adolescents (street children) |
| **SEXUALLY TRANSMITTED INFECTIONS** | 1.   Screening and case finding of STIs  2.   Early diagnosis and treatment of STI cases  3.   STI-prevention strategies  4.   Issues around antimicrobial resistance |
| **HIV/AIDS** | 1. Tracing and reengagement in care 2. Diagnostics that meet the specifications for use in LMICs, especially point-of-care diagnostics targeting lower levels facilities 3. Assessing ART adherence 4. Assessing frequency of care visits/refills 5. Task sharing of specimen collection and point-of-care testing 6. Integration of HIV and sexual reproductive health services 7. Assessing ART initiation outside the health facility 8. Generation of data on drug safety and efficacy for PLHIV 9. Assessing integration of HIV and diabetes and hypertension care 10. Developing and introducing new paediatric drug formulations for treatment of HIV/AIDS 11. Developing psychosocial interventions for adolescents & youth 12. Balanced integration of diagnostic services |

1.	WHO. *World Health Organization Global adult estimates*. 2020 [cited 2024; Available from: https://www.who.int/reproductivehealth/topics/rtis/STIs-Estimates.pdf.

2.	UNICEF. *Maternal mortality declined by 40 per cent between 2000 and 2023*. 2025; Available from: https://data.unicef.org/topic/maternal-health/maternal-mortality/.

3.	WHO. *Sexual and Reproductive Health*. 2022 2024]; Available from: https://www.who.int/reproductivehealth/congenital-syphilis-estimates/en/.

4.	Mbadugha, N. *Big Data and AI in Africa: Why the Future of the Continent Is Artificially Intelligent and Digitally Enabled*. NextBillion Guest Articles 2021 [cited 2021 Nov 23]; Available from: https://nextbillion.net/big-data-ai-africa-artificial-intelligence-digital/.

5.	Idele, P., et al., *Epidemiology of HIV and AIDS among adolescents: current status, inequities, and data gaps.* J Acquir Immune Defic Syndr, 2014. **66 Suppl 2**: p. S144-53.

6.	Bolarinwa, O., et al., *Sexual and reproductive health rights in the era of artificial intelligence.* Lancet, 2024. **404**(10448): p. 120-121.

7.	Bolarinwa, O.A., A. Mohammed, and V. Igharo, *The role of artificial intelligence in transforming maternity services in Africa: prospects and challenges.* Ther Adv Reprod Health, 2024. **18**: p. 26334941241288587.

8.	Harry, A., *The Future of Medicine: Harnessing the Power of AI for Revolutionizing Healthcare.* International Journal ofMultidisciplinary Sciences and Arts, 2023. **2**(1).

9.	El Arab, R.A., et al., *Artificial intelligence in nursing: a systematic review of attitudes, literacy, readiness, and adoption intentions among nursing students and practicing nurses.* Front Digit Health, 2025. **7**: p. 1666005.

10.	Emaojo, E. *How AI is widening the global/human development gap*. 2025; Available from: https://www.developmentaid.org/news-stream/post/196997/equitable-distribution-of-ai.

11.	McKenna, A. *sub-Saharan Africa*. 2026; Available from: https://www.britannica.com/place/sub-Saharan-Africa.

12.	della Chiesa, B., Scott, J, Hinton, C, *Languages in a Global World; LEARNING FOR BETTER CULTURAL UNDERSTANDING*. 2012.

13.	Bain, L., Njei, B., *Equitable intelligence for Africa: strengthening global health artificial intelligence (AI) governance.* Pan African Medical Journal, 2025. **52**(179).

14.	Frasier K, H.M., Werpachowski N, Fritts H., *The Blind Spots of Artificial Intelligence in Skin Cancer Diagnosis.* Dermis, 2025. **5**(2): p. 35.

15.	Oseku E, M.P., Semakula H, Kahuma CA, Balaba M, Naggirinya AB, King RL, Parkes-Ratanshi R, *Crowdsourcing a Training Dataset of Question-and-Answer Pairs for AI-Enabled Health Information Tools on Sexually Transmitted Infections: Protocol for a Cross-Sectional Exploratory Survey Study.* JMIR Res Protoc 2025. **14**(e70005).

16.	Union, A., *Continental Artificial Intelligence Strategy:*

*Harnessing AI for Africa’s Development and Prosperity*, A. Union, Editor. 2024.
