# Supplementary material for: Exploring Perceptions of Leveraging AI to Improve Outcomes in Maternal, Sexual, and Reproductive Health in Sub-Saharan Africa: Exploratory Qualitative Study
Source: J Med Internet Res. 2026 Aug 13;28:e84378. doi: 10.2196/84378 (PMC13472523; doi:10.2196/84378)
Supplement: Supplementary file 3 [file jmir-v28-e84378-s002.docx]

***Personal Characteristics of the Research Team***

| **INTERVIEWER/**  **FACILITATOR** | **GENDER** | **DATA COLLECTION CONDUCTED** | **OCCUPATION AT TIME OF STUDY** | **RELEVANT EXPERIENCE AND TRAINING** |
| --- | --- | --- | --- | --- |
| Dr Rachel King PhD, MPH, BA | Female | Scoping Workshop | Co-Principal Investigator | Over 20-years of designing, conducting, analysing and writing up social and behavioural research studies across various health sub-domains ranging from HIV, TB, digital health and Artificial Intelligence for Health. Has over 100 published peer-reviewed articles. |
| Prof Rosalind Parkes-Ratanshi | Female | Round Table Discussion | Co-Principal Investigator | Over 20-years’ experience in multi-site STI research, diagnostic innovation and digital health research in sub-Saharan Africa. As co-lead for Hub for AI in Maternal, Sexual Reproductive Health in SSA, she supports ethical AI in reproductive health. Has over 60 peer-reviewed articles on reproductive health and digital health published. |
| Dr Cecilia Akatukwasa PhD, MPH, BSc | Female | Scoping Workshop | Social Science Lead | Has training in psychology and human behaviour. Obtained a certificate in qualitative research methods from UCSF. Has 12-years’ experience in qualitative methodology spanning data collection like interviewing, observation and facilitating group discussions. |
| Moreen Nanyonjo PGD M&E, BAECON. | Female | Scoping Workshop | Monitoring and Evaluation Officer | Three years’ experience in public health Anti-Retroviral Therapy (ART) clinics collecting patient level data for ART initiation. Two years’ experience planning and conducting stakeholder workshops and synthesising and reporting workshop outcomes. |
| Joshua Beinomugisha | Male | Key Informant Interviews  CAB Focus Group Discussion | Research Assistant | 13-years’ experience in qualitative research is in areas of conducting key informant interviews (KIIs), IDIs, focus group discussions (FGDs), tool development, transcription, qualitative data analysis and writing.Training includes Good Clinical Practice, Responsible Conduct of Research, Project Management in Global Health, Research writing, Dedoose and Nvivo software |
| Jackie Ssemata | Female | Key Informant Interviews  CAB Focus Group Discussion | Team Lead, Social Science | Over ten years’ experience in neurobehavioral and social science research in malaria, HIV, child health and digital health. |
